# Incidence and Risk of Treatment-Related Mortality with mTOR Inhibitors Everolimus and Temsirolimus in Cancer Patients: A Meta-Analysis

**DOI:** 10.1371/journal.pone.0065166

**Published:** 2013-06-13

**Authors:** Wei-Xiang Qi, Yu-Jing Huang, Yang Yao, Zan Shen, Da-Liu Min

**Affiliations:** Department of Oncology, the Sixth People’s Hospital, Shanghai Jiao Tong University, Shanghai, China; University of Porto, Portugal

## Abstract

**Background:**

Two novel mammalian targets of rapamycin (mTOR) inhibitors everolimus and temsirolimus are now approved by regulatory agencies and have been widely investigated among various types of solid tumors, but the risk of fatal adverse events (FAEs) with these drugs is not well defined.

**Methods:**

We searched PubMed, EMBASE, and Cochrane library databases for relevant trials. Eligible studies included prospective phase II and III trials evaluating everolimus and temsirolimus in patients with all malignancies and data on FAEs were available. Statistical analyses were conducted to calculate the summary incidence, RRs and 95% confidence intervals (*CI*s) by using either random effects or fixed effect models according to the heterogeneity of the included studies.

**Results:**

A total of 3322 patients with various advanced solid tumors from 12 trials were included. The overall incidence of mTOR inhibitors associated FAEs was 1.8% (95%CI: 1.3–2.5%), and the incidences of everolimus related FAEs were comparable to that of temsirolimus (1.7% versus 1.8%). Compared with the controls, the use of mTOR inhibitors was associated with an increased risk of FAEs, with a RR of 3.24 (95%CI: 1.21–8.67, *p* = 0.019). On subgroup analysis, a non-statistically significant increase in the risk of FAEs was found according to different mTOR inhibitors, tumor types or controlled therapy. No evidence of publication bias was observed.

**Conclusion:**

With the present evidence, the use of mTOR inhibitors seems to increase the risk of FAEs in patients with advanced solid tumors. More high quality trials are still needed to investigate this association.

## Introduction

The mammalian target of rapamycin (mTOR) kinase is a key element of intracellular signal transduction, and responsible for the regulation of cell growth, survival, proliferation, and angiogenesis [1], [2], [3], [4]. In many tumor types, the mTOR pathway is found activated through several different underlying mechanisms [5], [6], [7], [8]. Since this pathway is believed to largely drive the malignant behavior of several of these tumors, mTOR-inhibition is considered an attractive means to apply as anti-tumor treatment [4], [9], [10]. The first identified mTOR inhibitor is rapamycin (sirolimus). Subsequently, the most potent rapamycin analogues (rapalogues), such as everolimus, temsirolimus and deforolimus, have been developed. During the past decades, several clinical trials testing the rapalogues in monotherapy or in combinations with other cytotoxic agents have been conducted in patients with many malignancies, and survival benefits have been observed in advanced renal cell cancer when compared to the controls [11], [12]. Based on these results, the United States Food and Drug Administration (FDA) have approved everolimus and temsirolimus as the treatment for advanced renal cell cancer [13]. In addition, mTOR inhibitors also show greatly anti-tumor effects for advanced breast cancer, mantle cell lymphoma (MCL) and pancreatic neuroendocrine tumors [14], [15], [16]. As a result, the use of mTOR inhibitors is expected to increase in the near future, and an appreciation for the toxicity profiles of mTOR inhibitors is thus urgently needed.

Fatal adverse events (FAEs) are defined as deaths that are usually secondary to the use of the pharmaceutical agent [17]. Patients with cancer may be at an increased risk because of the progressive nature of malignancy as well as the adverse events (AEs) profiles of chemotherapeutic agents. As a result, determining the incidence and risk of drugs related FAEs is important for closely monitoring and planning appropriate strategies to limit their effects. Recently, several meta-analyses of vascular epithelial growth factor (VEGF)-targeted agents have shown that the use of angiogenesis inhibitors is associated with a significant increase in the relative risk (RR) of FAEs compared with the controls [17], [18], [19]. As mTOR inhibitors also have indirect inhibitory effects on VEGF pathways, the use of these drugs might be also associated with an increased risk of FAEs [4], [20], [21]. Indeed, FAEs have occasionally been reported in clinical trials with mTOR inhibitors, although no significant and definitive data have been established. As a result, we conduct this meta-analysis of published prospective trials to determine the incidence and risk of mTOR inhibitors associated FAEs in patients with cancer.

## Methods

### Search strategy

Study was conducted according to the Preferred Reporting Items for Systematic Reviews and Meta-Analyses (PRISMA) statement (see checklist table S1) [22], [23]. We searched the Pubmed (data from 1966 to Dec 2012), EMBASE (data from 1980 to Dec 2012), and Cochrane library databases (up to Dec 2012) for relevant trials. The search was conducted by using the keywords “mTOR inhibitor”, “everolimus”, “temsirolimus”, “RAD001”, “CCI-779”, “randomized”, “cancer” and was limited to human studies and prospective clinical trials published in English. Abstracts presented at the annual meetings of the American Society of Clinical Oncology (ASCO) and the European Society of Medical Oncology (ESMO) (from 2001 to 2012) were also searched manually using the same keywords to identify relevant clinical trials; Additionally, we searched the clinical trial registration website (http://www.ClinicalTrials.gov) to obtain information on the registered randomized controlled trials (RCTs); however, only trials published in peer-reviewed publications, in full manuscript form, were included. Each publication was reviewed and in cases of duplicate publication only the most complete, recent, and updated report of the clinical trial was included in the meta-analysis.

### Study selection

The goal of this study was to determine the incidence of mTOR inhibitors associated FAEs and establish the association between treatment with mTOR inhibitors and the risk of FAEs. Thus, Phase I trials were omitted due to multiple dose level and limited sample sizes. Clinical trials that met the following criteria were included in the meta-analysis: (1) prospective phase II and III trials of patients with cancer; (2) participants assigned to treatment with mTOR inhibitor (alone or in combination); and (3) available data regarding events or incidence of FAEs and sample size. The quality of reports of clinical trials was assessed and calculated using the 5-item Jadad scale including randomization, double-blinding, and withdrawals as previously described [24].

### Data extraction and clinical endpoints

Data extraction was conducted independently by two investigators (Q.W.X. and S.Z.), and any discrepancy between the reviewers was resolved by consensus. For each study, the following information was extracted: author’s name, year of publication, trial phase, number of enrolled subjects, treatment arms, number of patients in treatment and control groups when available, underlying malignancy, median age, median treatment duration, median progression-free survival, adverse outcomes of interest (fatal adverse events), name and dosage of the mTOR inhibitors and the dosing schedules used. The primary end point FAE definition was treatment emergent, non-disease-related, fatal adverse events. FAEs were reported according to the National Cancer Institute’s Common Terminology Criteria for Adverse Events (CTCAE) criteria, version 2 or 3 [25]. Both versions are similar in defining fatal adverse events as grade five, though version three requires attribution to specific adverse events while version two did not have such requirements. We excluded events that were reported as related to disease progression, but included all events with unspecified attribution and included events regardless of attribution to treatment provided that they were not related to disease progression.

### Statistical analysis

For the calculation of incidence, the number of patients experiencing a FAEs and the total number of patients being treated with the study drug were extracted from the safety profiles of all the selected studies. For each study, we derived the proportion and 95% confidence interval (*CI*) of patients with FAEs. For studies with a control group in the same trial, we also calculated and compared the relative risk (RR) of FAEs. For one study that reported zero events in the control arm, we applied the classic half-integer correction to calculate the RR and variance [26]. Between-study heterogeneity was estimated using the χ^2^-based Q statistic [27]. Heterogeneity was considered statistically significant when *P*_heterogeneity_ <0.05 or *I* ^2^ >50%. If heterogeneity existed, data was analyzed using a random effects model. In the absence of heterogeneity, a fixed effects model was used. A statistical test with a *p*-value less than 0.05 was considered significant. We also conducted the following prespecified subgroup analyses: different mTOR inhibitors, tumor types and controlled therapy. The presence of publication bias was evaluated by using the Begg and Egger tests [28], [29]. All statistical analyses were performed by using Stata version 12.0 software (Stata Corporation, College Station, Texas, USA) and Open Meta-Analyst software version 4.16.12 (Tufts University, URL http://tuftscaes.org/open_meta/).

## Results

### Quantity and Quality of evidence

**Figure 1:**
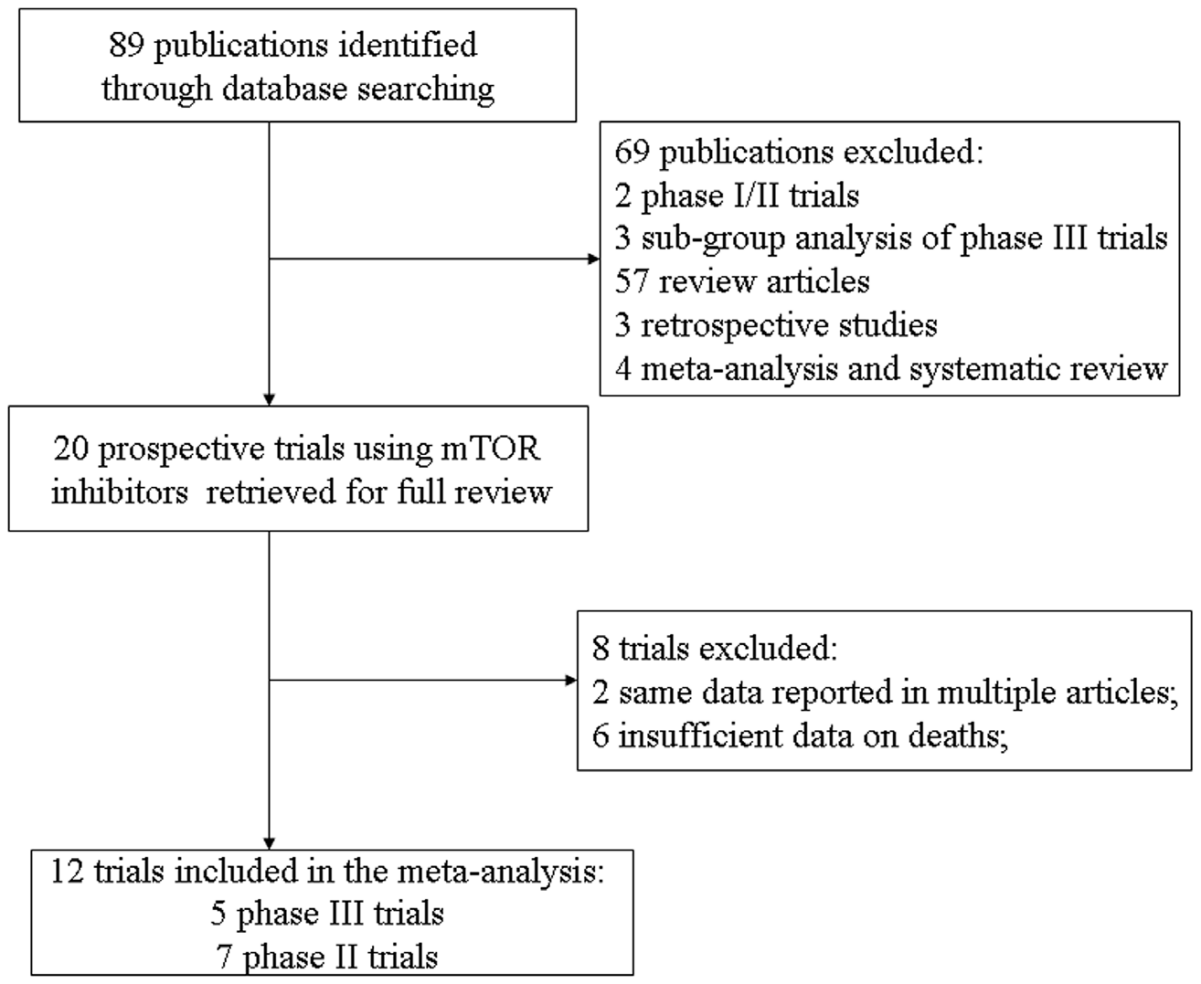
Flow chart of trial selection process in the meta-analysis.

Our study yielded a total of 89 potentially relevant abstracts from the literature. Sixty-nine studies were initially excluded for being Phase I trials, retrospective clinical trials, meta-analysis of randomized controlled trials (RCTs), and review articles. Subsequently, 8 trials were excluded for the following reasons: 2 trials were updated reports of previous trials [30], [31]; 6 trials did not have an adequate safety profile data listing for FAEs due to study drug. Finally, 12 prospective clinical trials, five phase III, and seven phase II trials, were selected for analysis. Figure 1 outlined the selection process in detail. These trials represented six studies with temsirolimus [11], [32], [33], [34], [35], [36], and six with everolimus [12], [14], [15], [37], [38], [39]. A total of 3322 patients were available for the meta-analysis, with 1015 patients from temsirolimus trials, and 2307 from everolimus trials. Four trials were performed in patients with RCC [11], [12], [32], [36], three in patients with breast cancer [14], [33], [38], two in patients with neuroendocrine tumors [15], [39], two in patients with SCLC [35], [37] and one in patients with advanced solid tumors [34]. The 12 included trials were published in 2004–2012. The median age of study participants was in the range of 55–68 years (some studies only reported the mean age). Sample sized were in the range of 24 to 724 patients, with eight trials including >100 patients each. Six trials were randomized controlled trials [11], [12], [14], [15], [38], [39], while the others were prospective phase II trials. The dosage of mTOR inhibitors significantly varies among included trials: temsirolimus was used ranging from 20 mg to 250 mg iv weekly, and the dose of everolimus was 10 mg or 5 mg po. daily, respectively. The quality of 12 included trials was high: five trials had Jadad scores of 5, which mentioned the concealment of allocation clearly in the randomization process, and provided the number of patients who withdrew from the trials. Four trials did not mention the blinding of allocation clearly in the randomization process, and did not mention the concealment of allocation, thus had Jadad scores of 2. Another three trials only provided the number of patients who withdrew from the trials, thus had Jadad scores of 1. The characteristics of each included trial were presented in table 1.

**Table 1:** Baseline characteristics of the 12 trials included in the meta-analysis (n = 3322).

Authors/year/phase	Histology	Patients enrolled	Treatment Arm	Median age (years)	Median treatment duration (months)	Median PFS/TTP (months)	Median OS (months)	No. for analysis	No.of FAEs	Jadad score
Atkins M.B. et al./2004/II	RCC	111	Temsirolimus 25mg. q.w.	55	NR	6.3	13.8	36	0	2
			Temsirolimus 75mg q.w.	58	NR	6.7	11.0	38	0	
			Temsirolimus 250mg q.w.	57	NR	5.2	17.5	37	0	
Chan S. et al/2005/II	MBC	109	Temsirolimus 75mg q.w.	55	11 weeks	9.9 weeks	NR	55	1	2
			Temsirolimus 250mg q.w.	56	12 weeks	14.3 weeks	NR	53	0	
Hudes G. et al/2007/III	RCC	626	Interferon	60	NR	1.9	7.3	200	1	2
			Temsirolimus 25mg q.w.	58	NR	3.8	10.9	208	2	
			Interferon plus Temsirolimus 15mg q.w.	59	NR	3.7	8.4	208	7	
Pandya K.J. et al/2007/II	SCLC	87	Temsirolimus 25 mg q.w.	61	NR	1.9	6.6	44	0	2
			Temsirolimus 30 mg q.w.	59	NR	2.5	9.5	41	0	
Motzer R.J. et al/2008/III	RCC	410	Everolimus 10 mg qd	61	141 days	4.0	NR	269	4	5
			Placebo	60	60 days	1.9	8.8	135	1	
BaselgaJ. et al/2009/II	Breast Cancer	270	Everolimus 10mg qd plus letrozole	68	NR	NR	NR	137	0	5
			Placebo plus letrozole	66.9	NR	NR	NR	132	0	
Tarhini A et. al/2010/II	SCLC	40	Everolimus 10mg qd	64	42 days	1.3	6.7	40	0	1
Xu B. et al/2011/II	Advanced solid tumors	24	Everolimus 5 mg qd	55	136.5 days	NR	NR	NR	0	1
			Everolimus 10mg qd	56	63.5 days	NR	NR	NR	0	
Yao J.C. et al/2011/III	NET	410	Everolimus 10mg qd	58	8.79	11.0	NR	204	7	5
			Placebo	57	3.74	4.6	NR	203	1	
Pavel M.E. et al/2011/III	NET	429	Everolimus 10mg qd plus octreotide LAR	60	37 weeks	16.4	NR	215	0	5
			Placebo plus octreotide LAR	60	36.6 weeks	11.3	NR	211	0	
Baselga J. et al/2012/III	MBC	724	Everolimus 10 mg qd plus exemestane	62	14.6 weeks	6.9	NR	482	7	5
			Placebo plus exemestane	61	12.0 weeks	2.8	NR	238	1	
Sun Y. et al/2012/II	RCC	82	Temsirolimus 20mg/m2 q.w.	58	NR	8.7	NR	6	1	1
			Temsirolimus 25mg q.w.	55	NR	7.3	19.8	76	0	

Abbreviations: PFS, progression-free survival; NET, neuroendocrine tumors; RCC, renal cell cancer; NSCLC, non-small-cell lung carcinoma; LAR: long-acting repeatable; MBC, metastatic breast cancer; SCLC, small-cell lung cancer; FAEs: fetal adverse events; NR, not reported.

### Pooled Analysis Results

**Figure 2:**
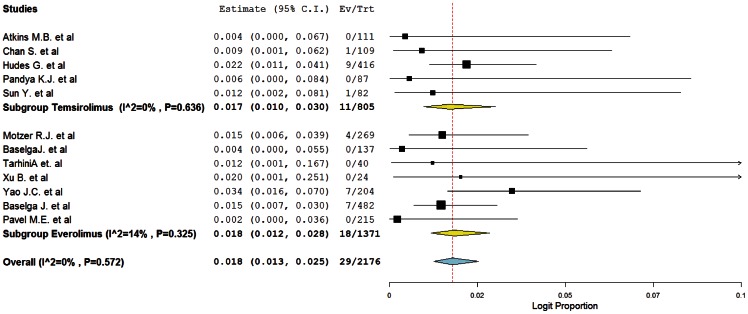
Forest plot for meta-analysis of incidence of FAEs in cancer patients assigned mTOR inhibitors.

For the incidence of FAEs, all mTOR inhibitors treatment arms were included, representing a total of 2176 patients. By using a fixed-effects model (heterogeneity test: *I* ^2^ = 0%; *p* = 0.572), the incidence of FAEs due to mTOR inhibitors was determined to be 1.8% (95%CI: 1.3–2.5%). The highest incidence (3.4%, 95%CI: 1.6–7.0%) was observed in a phase III trial of everolimus in patients with pancreatic neuroendocrine tumors [15]. No FAEs were observed in six trials [32], [34], [35], [37], [38], [39]. When stratified by each mTOR inhibitors, the incidence was 1.7% (95%CI: 1.0–3.0%) for temsirolimus, 1.8% (1.2–2.8%) for everolimus (figure 2).

**Figure 3:**
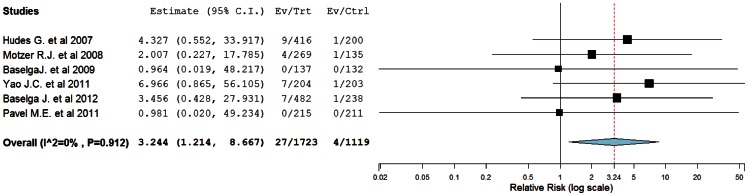
Relative risk of mTOR inhibitors associated FAEs versus control from randomized controlled trials of patients with cancer.

To investigate the specific contribution of mTOR inhibitors to the development of FAEs and exclude the influence of confounding factors such as underlying malignancy, and other therapeutic interventions, we therefore determined the relative risk (RR) of mTOR inhibitors associated FAEs. A total of 2912 patients from six trials were included for the analysis [11], [12], [14], [15], [38], [39]. The pooled RR for FAEs showed that the use of mTOR inhibitors significantly increased risk of developing FAEs in cancer patients with RR of 3.244 (95%CI: 1.214–8.667, *p* = 0.008, figure 3) using a fixed-effects model (*I* ^2^  = 0%, *p* = 0.912). Similar results were observed in subgroup analysis based on mTOR inhibitors: in five trials using everolimus as study drug (everolimus, n = 1307; placebo/controls, n = 919), the pooled results showed that there was a tendency to increase the risk of FAEs with RR of 2.98 (95% CI, 0.97 to 9.12; *P* = 0.056). Only one included trial using temsirolimus as study drug (temsirolimus, n = 416; placebo/controls, n = 200) also demonstrated a non-statistically significant increase in the risk of FAEs yielding an RR of 4.40 (95% CI, 0.55 to 34.98; *P = *0.16). To determine whether tumor type had an influence in the RR of FAEs with mTOR inhibitors, we performed a subgroup analysis according to tumor types. The combined results showed that the use of mTOR inhibitors had a tendency to increase the risk of developing FAEs among patients with renal cell cancer (RR, 3.01; 95% CI, 0.67 to 13.47; *P* = 0.15), breast cancer (RR, 2.00; 95% CI, 0.26 to 15.23; *P* = 0.50), and neuroendocrine tumors (RR, 2.00; 95% CI, 0.20 to 20.15; *P* = 0.56), although the difference was not statistically significant (table 2). Of note, the occasional wide variation in the CIs might indicate that the association of different mTOR inhibitors and tumor types with FAEs might be different, but there was lack of statistical power to demonstrate a significant difference. Additionally, we performed a sub-group risk analysis stratified according to controlled therapy. The combined results showed that the use of mTOR inhibitors was associated with a non-significantly increased risk of FAEs in comparison with placebo (RR 4.14, 95%CI: 0.97–17.64) or non-placebo therapy (RR 3.89, 95%CI: 0.90–16.86). Individual specified and non-specified causes of fatal adverse events were listed in table 3. Of the 29 fatal adverse events on the treatment arms and 4 fatal adverse events on the controlled arms, 55.2% and 75% were of non-specified etiology, respectively. Of those fatal adverse events that specified, the most common causes of FAEs were pneumonia (30.8%) and sepsis (38.5%), respectively. One possible explanation for this finding was that the use of mTOR inhibitors could cause non-infectious pneumonitis, although the mechanisms of mTOR related pneumonitis was still unknown. In accordance with our results, a recent meta-analysis conducted by Iacovelli R. et al [40] found that the incidence of all- and high-grades pneumonia in patients receiving mTOR inhibitors was 10.4% and 2.4%, respectively. And there was 31- and 8.8-folds increased risk of developing all- and high-grades pneumonia when compared to controls. Additionally, the mTOR inhibitors were potent immunosuppressants and showed strong effects on the B-cell functions, CD80 and CD86 expression, proliferation, IgG/IgM and cytokine production in a dose-dependent manner [41], [42]. As a result, the use of mTOR inhibitors was associated with increased risk of infection due to immunosuppression. No evidence of publication bias was detected for the incidence or the RR of FAEs in this study by either Begg or Egger’s test (RR of FAEs: Begg’s test *p* = 0.31; Egger’s test *p* = 0.40).

**Table 2:** Risk ratio of fatal adverse events associated with mTOR inhibitors.

Groups	No. of studies	No. of events/sample size		RR	95%CI	*P* value
		mTOR inhibitors	Placebo/control			
**Overall**	6	27/1723	4/1119	3.24	1.21–8.67	0.019
**Drug type**						
Everolimus	5	20/1307	3/919	2.98	0.97–9.12	0.056
Temsirolimus	1	7/416	1/200	4.40	0.55–34.98	0.16
**Tumor type**						
RCC	2	13/685	2/335	3.01	0.67–13.47	0.15
BC	2	7/619	1/370	2.00	0.26–15.23	0.50
NET	2	7/419	1/414	2.00	0.20–20.15	0.56
**Controlled therapy**						
Placebo	2	16/1250	2/781	3.89	0.90–16.86	0.069
Non-placebo	4	11/473	2/338	4.14	0.97–17.64	0.055

Abbreviations: RCC, renal cell cancer; BC, breast cancer; NET, neuroendocrine tumor.

**Table 3:** Fatal adverse events by specific type.

	Events on mTOR inhibitor arms	Events on control arms
**Unspecified**	16	3
**Pneumonia**	4	0
**Sepsis**	5	0
**Tumor hemorrhage**	1	0
**Cerebrovascular incident**	1	0
**Renal failure**	1	0
**Suicide**	1	0
**Myocardial infarction**	0	1
**Overall**	29	4

## Discussion

Although cytotoxic chemotherapy has still been the mainstay for cancer treatment, advances in the knowledge of tumor biology and the molecular pathways involved in cancer cell proliferation have ushered the age of molecularly targeted agents for cancer treatment [43], [44]. In contrast with traditional cytotoxic agents, these agents offer the promise of improved efficacy and a more favorable toxicity prolife. However, unique common side effect profile of these agents including hypertension, rashes, and metabolic abnormalities has also been reported in clinical trials [45], [46], [47], [48], [49], [50]. The incidence and management algorithms for those common side effects have been well defined in previous researches, but there is much more challenging to appreciate the uncommon, yet serious, toxicities associated with these drugs.

The meta-analysis is a powerful statistical tool to estimate the incidence and risk of those uncommon serious drug-related toxicities and this approach has been utilized to demonstrate an increased risk in treatment related mortality with bevacizumab and VEGFR-TKIs in previous researches [17], [18], [19]. To the best of our knowledge, this is the first meta-analysis to investigate the incidence and risk of FAE associated with the mTOR inhibitors everolimus and temsirolimus. Our meta-analysis included 3322 patients from 12 trials demonstrates the overall incidence rate of FAEs is 1.8% (95%CI: 1.3–2.5%), and there is a significant three-times increased risk of death with these agents. However, a non-significantly increased risk of mTOR inhibitor associated FAEs is observed in sub-group analysis according to the mTOR inhibitors, tumor types and controlled therapy, for which we suggest several possible explanations: the small number of events recorded; under-reporting of rare (<5%) adverse events; the fact that clinical trials are usually not designed specifically to address toxic events; and the small number of randomized controlled trials included.

As mTOR inhibitors find more clinical applications and are used to treat a more heterogeneous patient population than those found in clinical trials, efforts are still needed to limit the risk of FAEs. Patients receiving mTOR inhibitors should be carefully monitored for the evidence of infection, especially patients with underlying known chronic lung disease or risk factors of infection. What’s more, as the use of mTOR inhibitors could cause non-infectious pneumonitis, which is characterized by non-infectious, non-malignant, and non-specific inflammatory infiltrates [40], [51]. Therefore, high-resolution computed tomography scans might be performed for patients present with cough and/or dyspnoea and/or hypoxemia, and/or fever when receiving mTOR inhibitors [51]. In addition, previous researches have demonstrated that pneumovax is effective in preventing both influenza (in 70–80% of people) and pneumococcal infection (in 60–70% of people) [52], [53], thus it might be a potential effective therapy for preventing mTOR inhibitors related pneumovax in cancer patients. However, until now, there is no specifically designed study to investigate the role of pneumovax for these patients, and studies focus on this issue is still needed.

Besides antitumor properties, mTOR inhibitors, especially sirolimus (rapamycin), have been widely used as an immunosuppressant in solid organ transplantation to prevent immune-mediated graft rejection [54], [55]. Interesting, sirolimus-associated pneumonitis has also been observed in renal and heart transplant recipients [56], [57], [58], and two deaths in patients who received sirolimus after heart transplants have been reported [57], [58]. However, the overall incidence of treatment mortality associated mTOR inhibitors is very low, and the use of sirolimus in transplant recipients is safe and tolerable [59].

This meta-analysis has some limitations. First, determining whether FAEs are attributable to mTOR inhibitors is particularly difficult in our study. Despite recommendations in the CTCAE version three (and beyond), the attribution of fatal events to particular toxicities was lacking in the majority of studies. Some studies did not clearly differentiate disease-related from non-disease-related fatal events. The lack of consistent reporting likely, in part, reflects the real-world difficulties of assigning causality to patient deaths, when the precise cause of death is unknown, or the cause of death may be easily associated with either the disease under study or the treatment being explored (e.g., thromboembolic events). However, in the current analysis, identical rules were utilized for abstracting events on both the mTOR inhibitors and control arms (treatment emergent fatal adverse events that were not specifically attributed to disease progression) which should have impacted over- or under-reporting of events on the mTOR inhibitors and control arms equally. Second, the ability of this study to detect variants in the FAE rate on the basis of specific drug or malignancy was limited because of low statistical power. Given that the conserved mechanism of action and known toxicities among the two study drugs are similar, it is unlikely that significant differences in FAEs would arise between them if more studies were available for analysis. As more high-quality studies of mTOR inhibitors in different malignancies and clinical settings become available, further analyses could be preformed to confirm the trends observed here. Third, the process by which investigators attribute FAE causality is a variable practice since FAEs were not the primary end point of any of the included studies. In addition, a continuity correction of 0.5 subjects with an event is used, which may have slightly overestimated the actual event rate of individual trials. Fourthly, although FAEs are prospectively collected for each individual study, this analysis is retrospective, and there are potentially important differences among the studies, including differing tumor types, dosage and administration schedule of mTOR inhibitors, periods of study conduct and study investigators. All of these would increase the clinical heterogeneity among included trials, which also made the interpretation of a meta-analysis more problematic. Additionally, our study includes a mixed population of patients treated mTOR inhibitors-based combination therapy or mTOR inhibitors alone, and patients received placebo or non-placebo therapy were also included in our study. Therefore, the treatment design is not the same in all arms, and it might be another source of heterogeneity. Finally, it is not an individual patient data analysis, and meta-analyses based on published data tend to overestimate treatment effects compared with individual patient data analyses. In addition, it precludes a more comprehensive analysis such as adjusting for baseline factors and other differences that existed between the trials from which the data were pooled.

In summary, our study demonstrates that the use of mTOR inhibitors seems to increase the risk of FAEs in patients with advanced solid tumors, but one should be cautious when interpreting these results due to the limitations of our study. Additionally, as this class of drugs gains greater clinical use, clinicians should be aware of the risks of FAEs with the administration of mTOR inhibitors in solid cancer, and closely monitoring is recommended during the therapy.

## Supporting Information

Table S1 **PRISMA checklist.**
(DOC)
